# Evidence of endogenously produced hydrogen sulfide (H_2_S) and persulfidation in male reproduction

**DOI:** 10.1038/s41598-022-15360-x

**Published:** 2022-07-06

**Authors:** Hedvika Řimnáčová, Jiří Moravec, Miriama Štiavnická, Jiřina Havránková, Ladan Monsef, Petr Hošek, Šárka Prokešová, Tereza Žalmanová, Tereza Fenclová, Jaroslav Petr, Milena Králíčková, Jan Nevoral

**Affiliations:** 1 Biomedical Center in Pilsen, Faculty of Medicine in Pilsen, Charles University, Pilsen, Czech Republic; 2 Laboratory of Animal Reproduction, Department of Biological Sciences, Biomaterials Research Cluster, Bernal Institute, Faculty of Science and Engineering, University of Limerick, Limerick, Ireland; 3 Institute of Animal Science, Prague 10-Uhrineves, Czech Republic; 4 Department of Histology and Embryology, Faculty of Medicine in Pilsen, Charles University, Pilsen, Czech Republic

**Keywords:** Spermatogenesis, Post-translational modifications

## Abstract

Persulfidation contributes to a group of redox post-translational modifications (PTMs), which arise exclusively on the sulfhydryl group of cysteine as a result of hydrogen sulfide (H_2_S) action. Redox-active molecules, including H_2_S, contribute to sperm development; therefore, redox PTMs represent an extremely important signalling pathway in sperm life. In this path, persulfidation prevents protein damage caused by irreversible cysteine hyperoxidation and thus maintains this signalling pathway. In our study, we detected both H_2_S and its production by all H_2_S-releasing enzymes (cystathionine γ-lyase (CTH), cystathionine β-synthase (CBS), and 3-mercaptopyruvate sulfurtransferase (MPST)) in male reproduction, including spermatozoa. We provided evidence that sperm H_2_S leads to persulfidation of proteins, such as glyceraldehyde-3-phosphate dehydrogenase, tubulin, and anchor protein A-kinase. Overall, this study suggests that persulfidation, as a part of the redox signalling pathway, is tightly regulated by enzymatic H_2_S production and is required for sperm viability.

## Introduction

Reactive oxygen species, reactive nitrogen species and reactive sulfur species (RONSS) are no longer considered harmful molecules leading to oxidative stress and apoptosis but are considered essential signalling molecules involved in many physiological events, such as sperm development, maturation, and capacitation^1–3^. Although a strong imbalance in redox reactions leads to damage and degradation of biomolecules, one molecule handles these conditions surprisingly well. Indeed, cysteine, a common amino acid incorporated into proteins, is the main player in the establishment of protein structure and antioxidant defence due to its sulfhydryl group (–SH) and its alternative modifications. In particular, the scavenging of RONSS through redox post-translational modifications (PTMs) of cysteine benefits the protein lifespan. Cysteine residues can be easily oxidized, and their oxidation is mostly reversible, which makes redox PTMs of cysteine unique signalling molecules. Moreover, these modifications often depend on each other, and one PTM leads to another, suggesting cross-regulation between individual redox PTMs^4,5^. For example, antioxidant enzymes are usually regulated by redox PTMs of cysteine under stress conditions. While S-nitrosylation (–SNO) and S-sulfenylation (–SOH) of –SH activate enzymes, irreversible hyperoxidation to sulfinic (–SO_2_H) and sulfonic (–SO_3_H) acids deactivates them. Additionally, proteins are rescued from irreversible hyperoxidation due to the reduction of SNO/SOH by glutathione (GSH) to form S-glutathionylation (–SSG)^6,7^. Persulfidation (–(S)_n_H) has the same rescue effect and thus plays an indisputable role in protein signalling across many tissues^8,9^. Accordingly, several proteins important for sperm physiology have been reported as –SNO targets^2,10^. Since –SNO could serve as a –(S)_n_H precursor, we can assume that –(S)_n_H will affect the activity of the proteins. Interestingly, no one has detected persulfidated proteins in spermatozoa, and the role of –(S)_n_H in male reproduction remains elusive. Although certain PTMs promote persulfidation of cysteine, this modification requires the action of hydrogen sulfide (H_2_S). H_2_S is physiologically produced in various cells by three specific enzymes from cysteine and homocysteine: cystathionine γ-lyase (CTH), cystathionine β-synthase (CBS), and 3-mercaptopyruvate sulfurtransferase (MPST). Notably, cysteine is the main target of H_2_S action and an important source of H_2_S. All these facts indicate that cysteine has a very unique role in H_2_S metabolism. Although H_2_S-releasing enzymes were detected in mouse testes^11^ and CBS and CTH in human spermatozoa^12^, complete knowledge about their distribution through mammalian spermatozoa is lacking.

Taken together, the results of previous studies indicate that endogenous production of H_2_S is associated with antioxidant defence and antiapoptotic and antiaging events reported in many tissues^13^. Unfortunately, studies on testicular tissue and spermatozoa are limited by the artificial supply of H_2_S rather than real H_2_S production^11,12,14,15^. Therefore, recent findings are unclear, and there are different conclusions depending on the donor concentration used. Although endogenous H_2_S production has been overlooked in male reproduction, these studies suggest that it has potential in reproductive physiology and deserves further attention. Our study provides the first evidence of physiological H_2_S production in spermatozoa and physiological contributions in the form of persulfidation in sperm physiology.

## Results

### Redox PTMs of cysteine do not drive maturation of male reproduction

**Figure 1:**
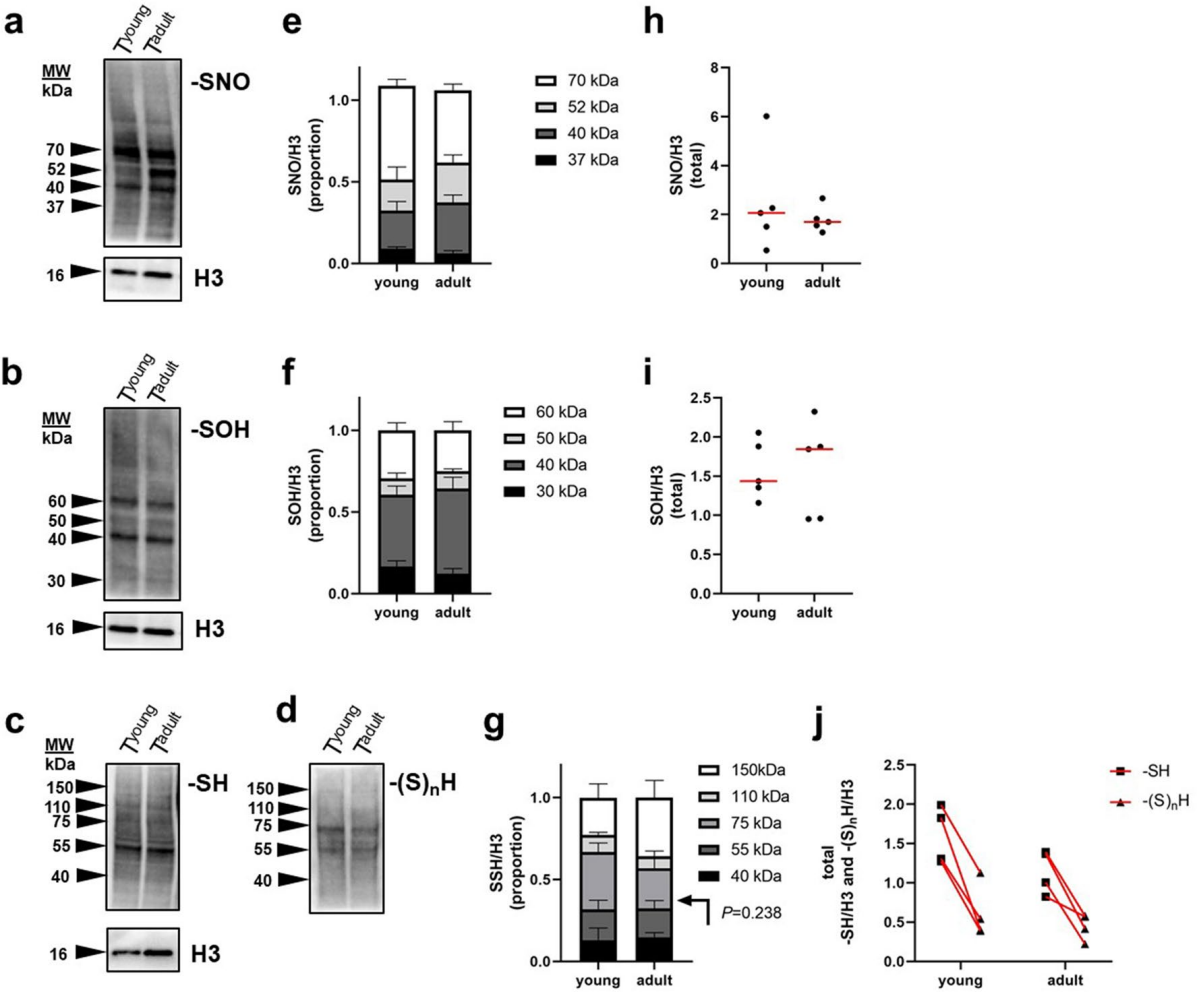
Detection of S-nitrosylation (–SNO), S-sulfenylation (–SOH), free thiols (–SH), and persulfidation (–(S)_n_H), redox PTMs of proteins, in young (i.e., prepubertal) and adult mouse testes. (**a**–**d**) Western blot detection of SNO, –SOH, –SH and –(S)_n_H. (**e**–**g**) Densitometric analysis of abundant bands. (**h**–**j**) Total density of abundant bands is expressed and young and adult males were compared; each dot represents an individual. (**j**) –SH and –(S)_n_H belonging to the same individual are connected by a red line. Histone 3 (H3) was used as a housekeeping internal control.

In this experiment, we focused on persulfidation in the broad context of other redox PTMs (–SNO and –SOH), in which cross regulations with persulfidation have been reported^4,5^. Due to the physiological contribution of cysteine PTMs in sperm maturation, we assumed that redox PTMs control the onset of spermatogenesis and thus drive sexual maturity in males. Therefore, we compared testicular lysates from mouse males before puberty onset, 21-day-old (young) and fully matured, 12–14-week-old males (adult) by Western blot (WB) detection of cysteine modifications: –SNO, –SOH, and –(S)_n_H (Fig. 1). We did not find any differences between the young and adult groups in (i) protein distribution (Fig. 1a–d), (ii) individual band intensity (Fig. 1e–g) or (iii) the total protein intensity (Fig. 1h–j) in any of the following parameters, suggesting that redox PTMs do not drive male reproductive maturation. The WBs of each PTM showed a specific ladder of bands (Fig. 1a–d). There was no band detected concurrently for –SNO/–SOH and –(S)_n_H, which suggests that there are different abundant proteins undergoing the specific PTMs, e.g., –SNO, –SOH, or –(S)_n_H. For instance, 55- and 75-kDa bands (Fig. 1d) were not detected, nor was –SNO or –SOH as intense as –(S)_n_H. This observation indicates that H_2_S is able to react and modify most –SNOs and –SOHs of certain proteins to form –(S)_n_H. –(S)_n_H was found in a small number of proteins compared with the detected sulfhydryl groups (–SH) (Fig. 1c,j). Thus, –(S)_n_H apparently does not drive male maturation, and it modifies exclusive proteins regardless of the maturity of testicular tissue.

### Persulfidation is abundant in testes compared to other tissues

**Figure 2:**
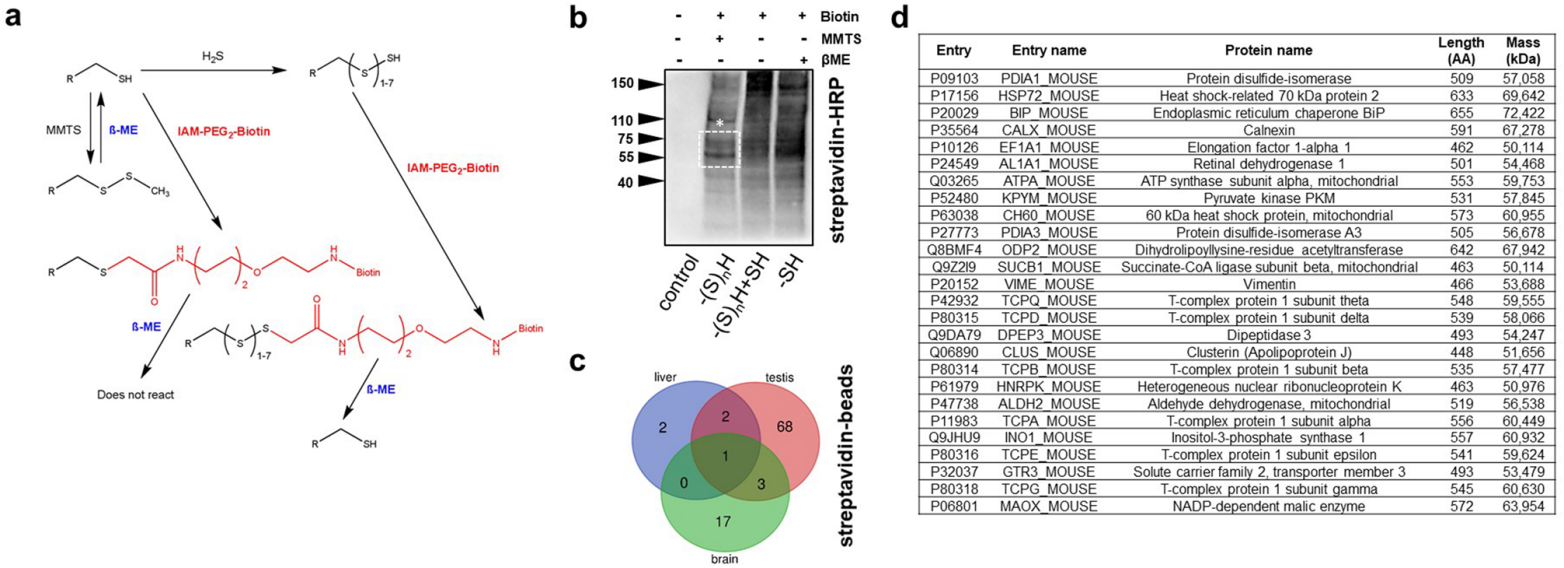
Persulfidation in mouse tissues with emphasis on testis. (**a**) Principle of selective detection of persulfidation (–(S)_n_H) using the thiol-selective binding ability of S-methyl methanethiosulfonate (MMTS) and the binding affinity of IAM-PEG-biotin to thiols (–SH). (**b**) β-Mercaptoethanol (β-ME), a reducing agent, was used to eliminate persulfide-biotin bonds and selectively detect free thiols. Bands in the size range 55–75 kDa (*) belonging to abundant proteins modified by –(S)_n_H. (**c**) Alternatively, selectively labelled –(S)_n_H with IAM-PEG-biotin was loaded on streptavidin-coated agarose beads. The eluted native proteins were digested and detected by nano-LC–MS. Liver, brain, and testicular tissues were processed via pulldown assays and nano-LC–MS detection, and persulfidated proteins were compared and expressed via Venn diagram. (**d**) Proteins of 55–75 kDa are presented in the table.

Persulfidation (–(S)_n_H) plays a plausible role in ageing, apoptosis and stress defence in many tissues, but information about this PTM in male reproduction is still missing. To shed light on this issue, we performed quantitative and qualitative analyses of –(S)_n_H in the testis. Because no specific maturity-dependent protein pattern was observed, the index of –(S)_n_H was compared in different kinds of tissues. We selectively labelled –(S)_n_H, accordingly with^16^ with slight modifications, while free –SH groups were blocked by MMTS and subsequently the –(S)_n_H groups were alkylated by IAM-PEG-biotin (Fig. 2a). Biotin was detected by streptavidin conjugated with horseradish peroxidase via WB detection (Fig. 2b). We observed that –(S)_n_H ranged from 40 to 150 kDa in the testes of adult mice. To validate the specificity of the method used, we prepared three specifically treated groups to detect: (S)_n_H + SH (no MMTS treatment), –(S)_n_H only (MMTS-treated), and naturally biotinylated proteins (nonalkylated control). The detected persulfidated proteins were then identified using pulldown assays and nano-LC–MS (Fig. 2c,d). We compared persulfidated proteins from the testis with those of the brain and liver, in which –(S)_n_H was previously widely described (Fig. 2c). We found proteins that were conservatively persulfidated across the tissues, but we found 68 proteins that were persulfidated only in the testis. Figure 2d represents persulfidated proteins specifically found in testes in the size range 55–75 kDa (bands in white rectangle marked with * in Fig. 2b). These findings suggest that –(S)_n_H targets proteins specifically in the testis, although these are widely expressed proteins.

### H2S-releasing enzymes are present in germ cells independent of their maturation stages in mouse testes

**Figure 3:**
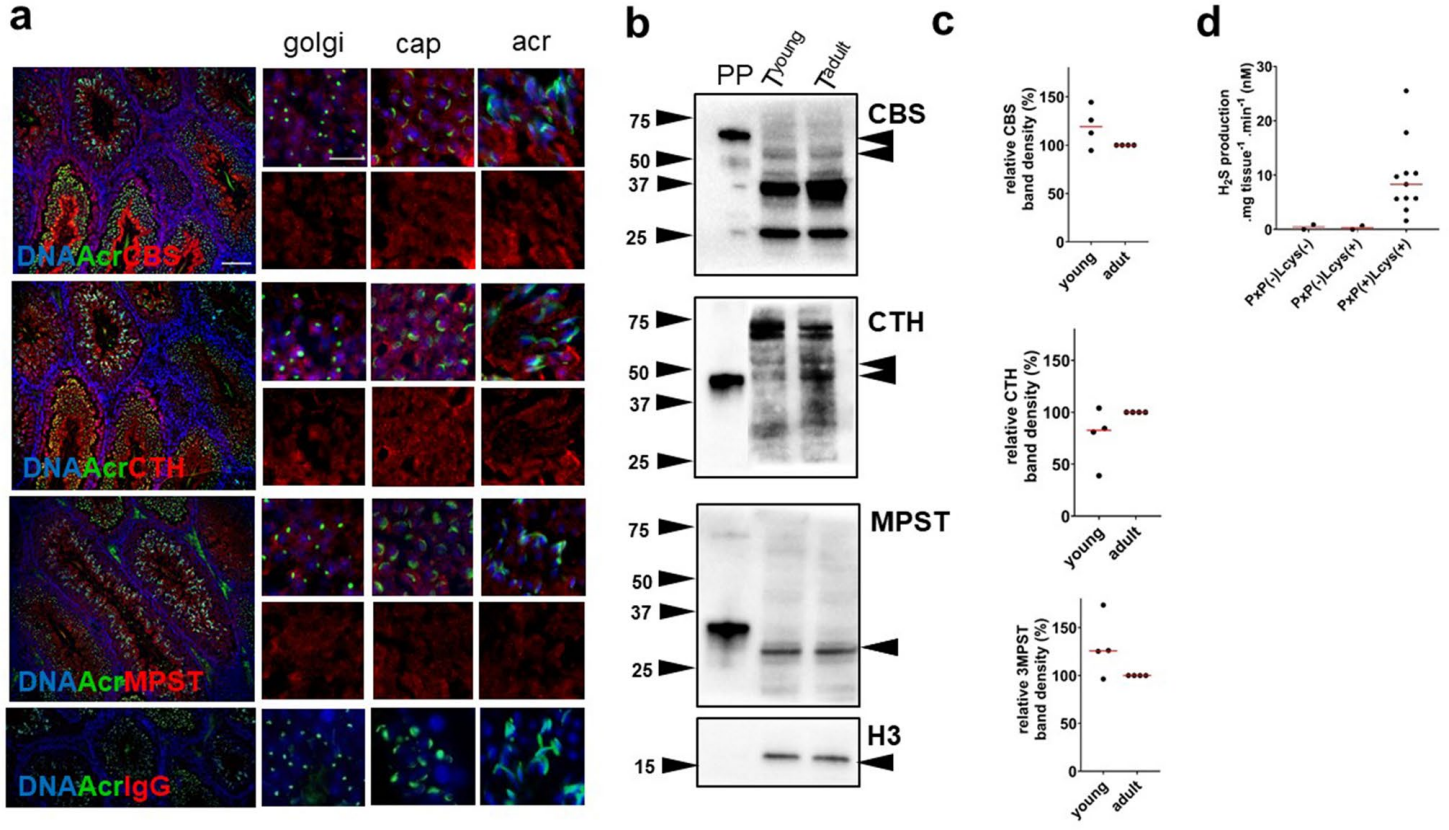
Detection of H_2_S-releasing enzymes in mouse testis: (**a**) immunofluorescence of testis sections. Acrosomal development stages of spermatids were recognized using PNA (200×). Individual stages of acrosomal biogenesis, representing the Golgi, cap, and acrosomal (Acr) stages, are emphasized (1000×). Scale bar 100 µm. (**b**) Cystathionine β-synthase (CBS), cystathionine γ-lyase (CTH) and 3-mercaptopyruvate sulfurtransferase (MPST) were detected by Western blot. (**c**) The comparison of prepubertal (young) and adult males was performed. (**d**) Colorimetric detection of H_2_S production in testes, which increased after the addition of pyridoxal-5′-phosphate (PxP) and l-cysteine.

We found that persulfidation (–(S)_n_H) is relatively abundant in the testes compared to the frequently studied liver and brain. –(S)_n_H is a well-known result of H_2_S action, and it is released enzymatically inside the cell. Therefore, we consider monitoring H_2_S production to be essential (Fig. 3). CBS, CTH and MPST have been previously detected in mouse testes^11^, but their distribution across developmental stages of germ cells has not been determined. Therefore, immunofluorescence detection of CBS, CTH, and MPST in testis sections was performed, depending on the developmental stages of germ cells within the seminiferous epithelial cycle. The results showed a strong dependency of enzymes on the cytoplasm of developing germ cells, regardless of the cell type and phase of spermiogenesis, distinguished in the Golgi, cap, and acrosomal stage (Fig. 3a). Enzyme independence of germ cell maturation was confirmed by WB performed on prepubertal and adult mice (Fig. 3b,c). The observation that H_2_S is not apparently associated with maturity level supports the versatility of H_2_S action in a cell. To elucidate H_2_S enzymatic production in testicular tissue, we performed colorimetric H_2_S detection (Fig. 3d). After the addition of pyridoxal-5′-phosphate (PxP), a cofactor of CBS and CTH, and l-cysteine, the substrate of enzymes, into the testis lysate, the production of H_2_S increased. To the best of our knowledge, we are the first to describe the relationship among H_2_S appearance, the enzymes responsible for its production, and the –(S)_n_H of proteins of male reproduction. Moreover, the association of H_2_S-releasing enzymes in germ cells predicts H_2_S production in fully differentiated spermatozoa and the possible role of –(S)_n_H in sperm physiology.

### Enzymatic production of H_2_S leads to persulfidation of protein cysteine in mouse spermatozoa

**Figure 4:**
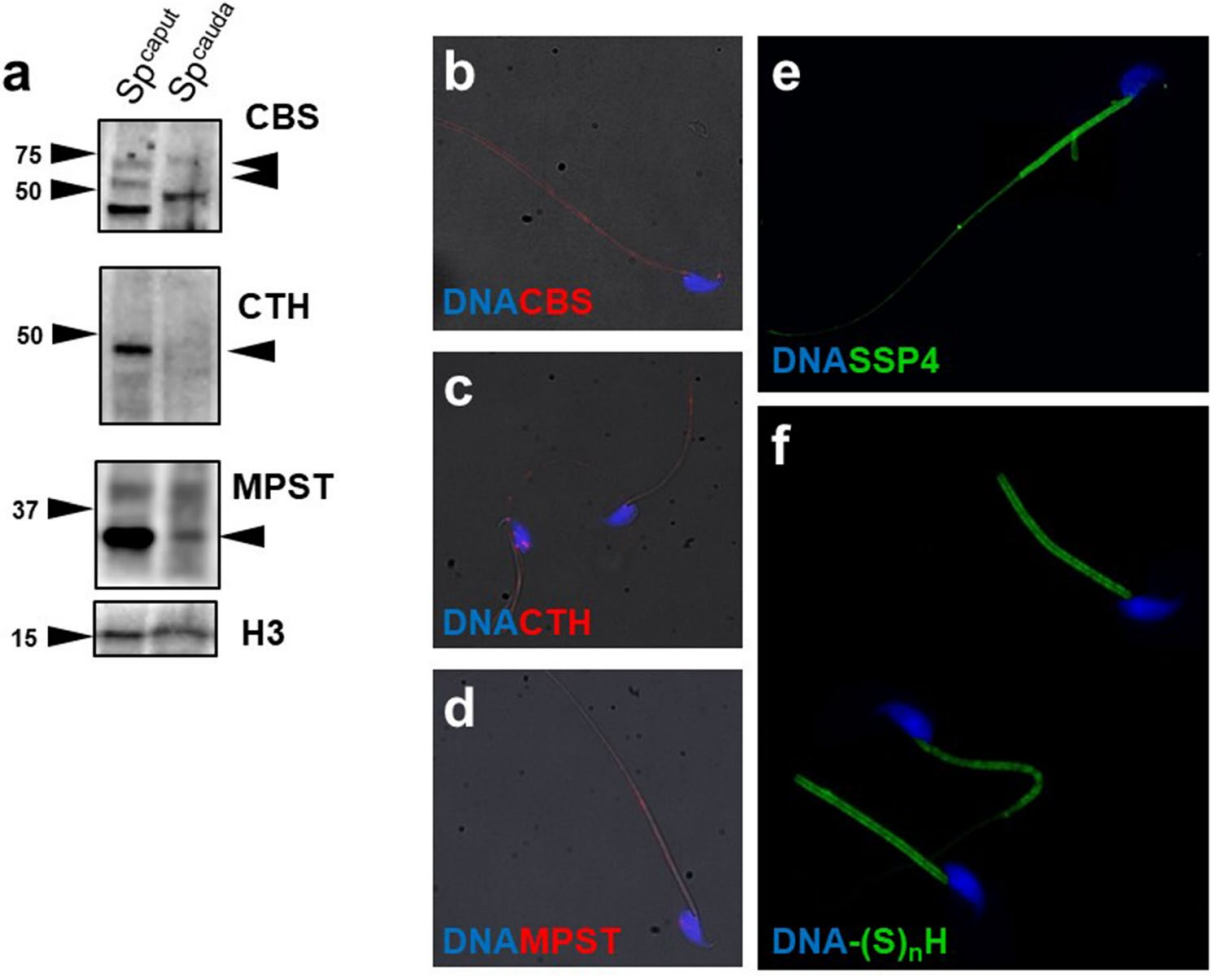
Production of H_2_S, its enzymes and persulfidation (–(S)_n_H) in mouse spermatozoa. (**a**) Western blot detection of cystathionine β-synthase (CBS), cystathionine γ-lyase (CTH) and 3-mercaptopyruvate sulfurtransferase (MPST) in mouse spermatozoa during their maturation in the epididymis. (**b**–**d**) Immunocytochemistry of CBS, CTH and MPST. (**e**) Localization of H_2_S production by Sulfane Sulfur Probe 4 (SSP4) and (**f**) –(S)_n_H. Spermatozoa were magnified (1000×).

The aim of this experiment was to examine H_2_S-releasing enzymes in mouse spermatozoa during their passage through the caput into the cauda epididymis. For analysis of H_2_S production, we compared the pattern of H_2_S-releasing enzyme subcellular distribution with H_2_S fluorescence visualization and protein persulfidation (–(S)_n_H) in fully differentiated spermatozoa. First, we detected CBS, CTH, and MPST via WBs in mouse spermatozoa from the caput epididymis (Sp^caput^) and cauda epididymis (Sp^cauda^) (Fig. 4a). Caput spermatozoa showed a strong signal of all H_2_S-releasing enzymes, whereas caudal spermatozoa showed either decreased (CBS), weak (MPST) or almost no signal (CTH) detected by WBs. To enhance the observation of H_2_S-releasing enzymes in caudal spermatozoa, we performed immunocytochemistry of single sperm cells (Fig. 4b–d). The signal of all enzymes along the entire length of the flagella was observed in caudal spermatozoa. Their enzymatic action was proved by H_2_S labelling by specific Sulfane Sulfur Probe 4 (SSP4) (Fig. 4e). Similar to H_2_S-releasing enzymes, the signal was emitted in the entire length of the flagella with the highest intensity in the midpiece. The observation of H_2_S production corresponding to H_2_S-releasing enzyme locations strongly supports the occurrence of H_2_S enzymatic production. Finally, we detected –(S)_n_H and found it exclusively in the midpiece (Fig. 4f). Although –(S)_n_H showed a slightly different pattern than H_2_S-releasing enzymes, it perfectly followed the site of the highest occurrence of H_2_S production. Therefore, we identified the midpiece as the location of H_2_S enzymatic activity, H_2_S production, and biochemical action.

### Sperm H_2_S-releasing enzymes produce H_2_S in spermatozoa across mammalian species

**Figure 5:**
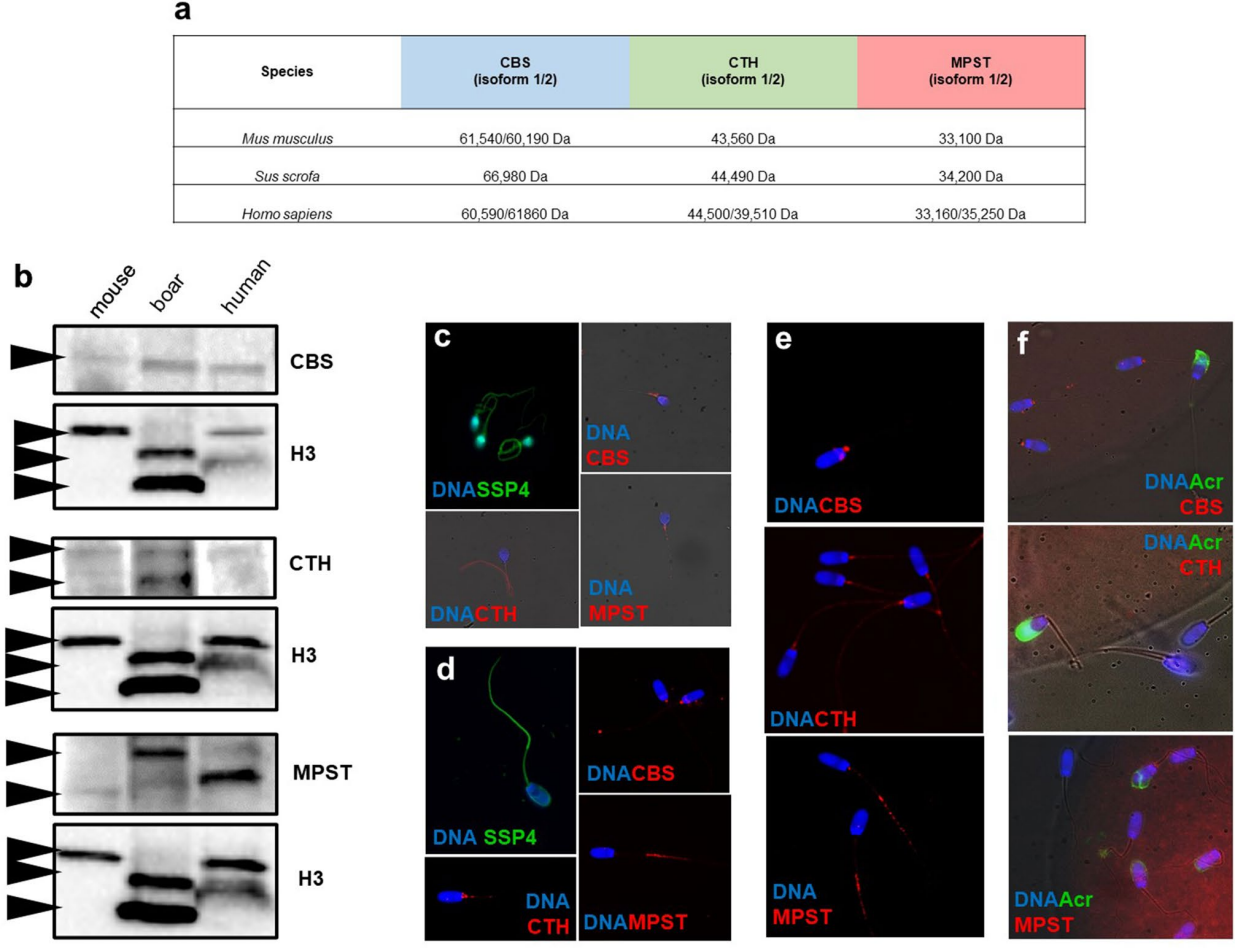
Interspecies production of H_2_S-releasing enzymes and H_2_S. (**a**) Species-specific molecular weight of the individual enzymes. (**b**) Western blot detection of CBS, CTH and MPST in human, boar and mouse spermatozoa. (**c**) Immunocytochemistry of all H_2_S-releasing enzymes and detection of H_2_S production by the SSP4 probe in human and (**d**) boar spermatozoa. (**e**) Decreases in CBS, CTH and MPST signal intensity during the last steps of boar sperm maturation, capacitation and (**f**) acrosomal reaction during sperm-*zona pellucida* binding. Spermatozoa were emphasized (1000×).

Based on previous findings of H_2_S production and the expression of H_2_S-releasing enzymes in mouse testes and spermatozoa, we suggest that H_2_S is enzymatically produced in spermatozoa across mammalian species. Therefore, we detected CBS, CTH, and MPST in human spermatozoa and in mouse and pig spermatozoa, the most common mammalian models. Enzyme detection was followed by elucidation of the release of SSP4-labelled H_2_S. Based on the known species-specific molecular weight of the individual enzymes based on the UniProtKB Database (Fig. 5a, see the phylogenetic trees in Supplementary Fig. S1), we identified all enzymes via WBs (Fig. 5b, see Supplementary Fig. S2 for whole WB membrane). Although immunocytochemistry of H_2_S-releasing enzymes showed interspecies variability in subcellular distribution, H_2_S production (SSP4) is quite constant in humans (Fig. 5c) and boars (Fig. 5d). This finding suggests that there is a different composition of the H_2_S-releasing enzymes responsible for most H_2_S production in a species-dependent manner. While H_2_S production colocalized with CTH in human spermatozoa, in boar spermatozoa, it colocalized instead with MPST. Sequential disappearance of H_2_S-releasing enzymes through spermatozoa maturation was shown by the evoked capacitation (Fig. 5e) and zona pellucida-binding assays of boar spermatozoa undergoing the acrosomal reaction, the last step of sperm maturation (Fig. 5f). This result complements our previous finding that H_2_S-releasing enzymes gradually decrease from spermatozoa during their maturation in the epididymis (Fig. 4a). Based on these observations, we conclude that the presence of H_2_S-releasing enzymes is partially lost from the cytoplasmic membrane during remodelling, which accompanies sperm maturation. Therefore, these enzymes do not appear to be involved in the sperm fertilization of eggs.

### Distribution and identification of persulfidates in human spermatozoa

**Figure 6:**
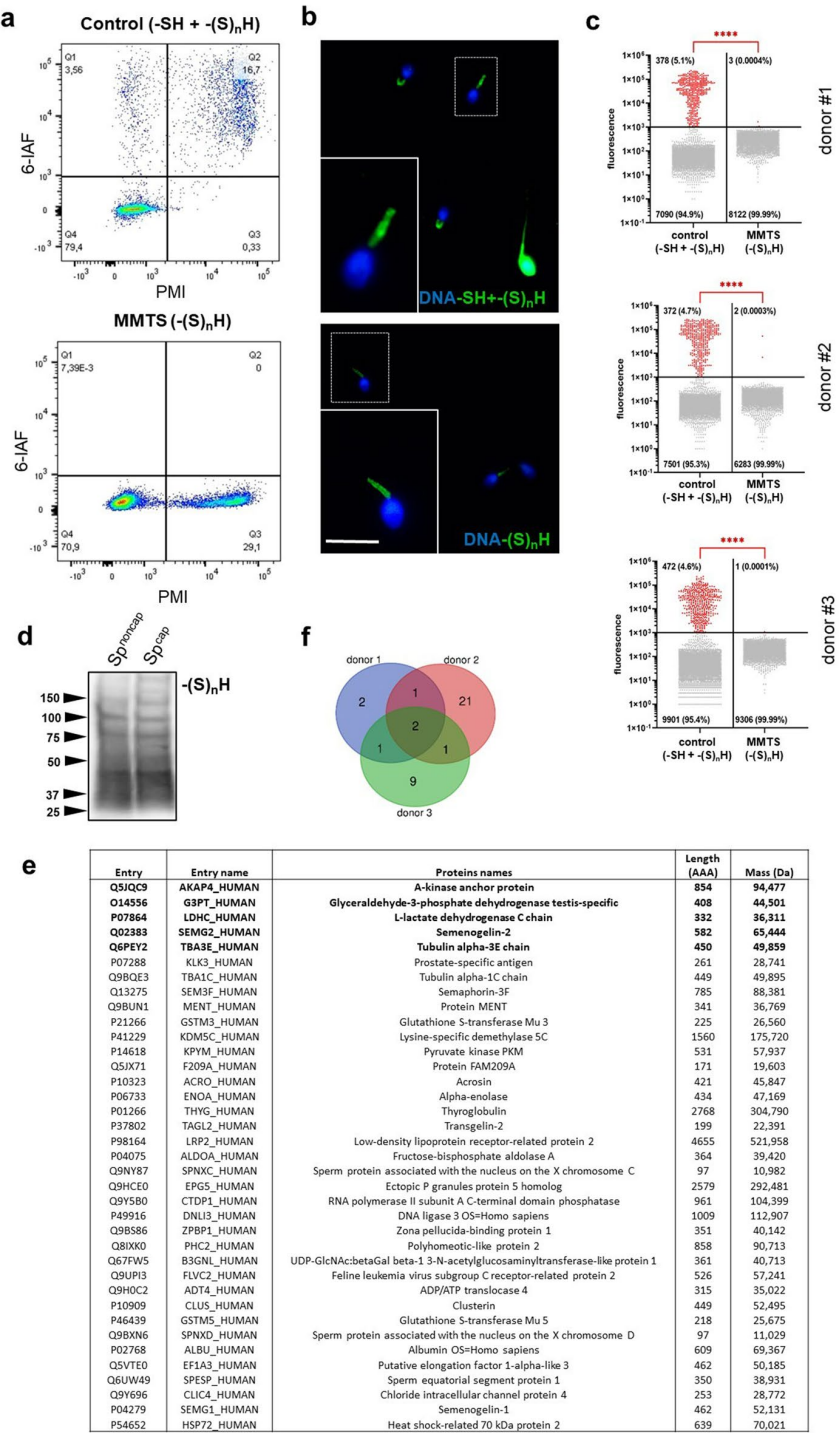
Free thiols and persulfidation analysis of proteins in human spermatozoa. (**a**) Flow cytometry of thiols (–SH) and persulfidation (–(S)_n_H) in spermatozoa due to 6-iodoacetamidofluorescein (6-IAF) staining without (control) and with (MMTS) blocking of free thiols. The dot plot shows the separation of sperm subpopulations according to plasma membrane integrity (PMI) and 6-IAF signal intensity. (**b**) Representative images of –SH and –(S)_n_H detection in situ showing 6-IAF staining patterns in the MMTS and control groups. The white rectangle indicates the –(S)_n_H-assumed signal in the midpiece of emphasized spermatozoa (scale bar: 10 μm). Spermatozoa were emphasized (1000×). (**c**) Presence of –SH + –(S)_n_H in the control and –(S)_n_H in the MMTS groups in live spermatozoa of three donors. Differences between the fluorescent signals belonging to –SH + –(S)_n_H and –(S)_n_H. Dots represent the fluorescence signal of individual spermatozoa. Lines express the mean of measured spermatozoa tested on live sperm subpopulations using the one-sample Wilcoxon test (****, *P* < 0.0001). (**d**) Persulfidated proteins separated by molecular weight in human spermatozoa being noncapacitated (Sp^non-cap.^) or capacitated (Sp^cap^). (**e**) All detected persulfidated proteins are shown in the table. Persulfidated proteins that matched at least in two donors are shown in bold. (**f**) Venn diagram shows 37 persulfidated proteins identified in three independent donors by nano-LC–MS.

In accordance with the aforementioned presence of H_2_S-releasing enzymes and H_2_S, we investigated the effects of H_2_S on persulfidation (–(S)_n_H) in the sperm protein of three normozoospermic donors using the approach described above. Concurrently, all three samples were subjected to flow cytometry analysis to obtain an overview of persulfidation occurrence across the entire sperm population. We observed –SH and –(S)_n_H with regard to plasma membrane integrity (PMI) using flow cytometry (Fig. 6a). In the control groups, spermatozoa were separated into three subpopulations according to susceptibility to PMI and 6-IAF staining as follows: the 1st quadrant (Q1) was live spermatozoa highly positive for 6-IAF, the 2nd quadrant (Q2) was dead spermatozoa highly positive for 6-IAF and the 4th quadrant (Q4) was live spermatozoa slightly positive for 6-IAF. When free thiols (*i.e.,* –SH) were specifically blocked in the MMTS experimental group, most 6-IAF signals disappeared, and spermatozoa moved to the less intense 6-IAF quadrants Q3 (death) and Q4 (live). This observation was supported by the detection of –SH and –(S)_n_H in situ, occurring in whole sperm or exclusively in the midpiece in the control (Fig. 6b). Following free thiol blocking, the sperm head-rich signal disappeared. For both flow cytometry and in situ detection, cell death was noticeably accompanied by substantial thiol exposure through the spermatozoon, while persulfidation was stably localized in the midpiece independent of live or dead sperm status. This observation was supported by the analysis of three independent semen donors, while the analysis was performed on the subpopulation of live spermatozoa (*i.e.,* Q1 and Q4 quartiles). There was a small population of spermatozoa (4.6–5.1%) showing high signal intensity belonging to –SH and –(S)_n_H in the control group. When –SH was blocked, only –(S)_n_H remained, and significantly weaker signals were detected in the sperm population in the MMTS group (Fig. 6c). Using the biotin-switched detection of –(S)_n_H in sperm lysate, we found that the abundance of persulfidated proteins did not show any capacitation-dependent difference (Fig. 6d), similar to our findings achieved in other mammalian models (Supplementary Fig. S3). Concurrently, these persulfide-labelled samples were subjected to pulldown assays, followed by nano-LC–MS peptide detection. We identified 37 persulfidated proteins with 99% confidence, in most cases being in a donor-specific pattern (Fig. 6f). Five proteins were found to match at least in two donors, marked in bold in the table containing all characterized persulfidated sperm proteins (Fig. 6e). Altogether, nano-LC–MS findings of midpiece-occurring proteins are in accordance with the H_2_S-releasing enzyme distribution, H_2_S labelling and in situ detection of persulfidation, underlining the spatiotemporal requirement of H_2_S activity in target protein modulation.

## Discussion

The sulfhydryl group (–SH) of cysteine provides a unique signalling pathway in which many redox molecules are involved. These molecules, such as nitric oxide (NO), hydrogen peroxide (H_2_O_2_) or hydrogen sulfide (H_2_S), oxidize or reduce –SH to form various redox post-translational modifications (PTMs) that control protein activity. These redox PTMs are unstable and continuously replacing each other on cysteine and together creating sophisticated signalling pathways. Persulfidation (–(S)_n_H) plays a central role in this pathway, as it replaces S-nitrosylation (–SNO) and S-sulfenylation (–SOH) on cysteine and thus regulates not only protein activities but also prevents their inactivation by hyperoxidation. There are many proteins that are regulated by redox PTMs in male reproduction^2,10^ but –(S)_n_H has not been investigated. In this study, we identified the existence of –(S)_n_H and its relation to endogenous H_2_S production and H_2_S-releasing enzyme localization in male reproduction with a focus on spermatozoa.

To the best of our knowledge, our study is the first to identify the protein –(S)_n_H in mouse testes and human spermatozoa. Subsequently, we compared persulfidated (–(S)_n_H) proteins found in mouse testes with –(S)_n_H from previously widely studied tissues, the brain and liver (Fig. 2c). Surprisingly, the largest amount of –(S)_n_H was detected in the testis, and 68 of the proteins were persulfidated uniquely in the testis (Fig. 2d). Although these proteins are not exclusive to the testes, they are apparently persulfidated in the testis only. It is well known that –(S)_n_H is the result of H_2_S action, and partially due to this effect, artificial supplementation of H_2_S has antiaging and antioxidant effects in many tissues, including spermatozoa^11,12,14^. There are several publications addressing H_2_S donor efficacy, although supplementation with exogenous H_2_S has possible toxic effects^15^. In contrast to these publications, there is no evidence about physiological endogenous H_2_S production and its consequences for male reproduction. Although all H_2_S-releasing enzymes have been previously detected in mouse testis^11^ and CBS and CTH in human spermatozoa^12^ and rat epididymis^17^, we immunodetected all three responsible enzymes in mouse testicular cross-sections and in spermatozoa of three mammalian species. Therefore, we claimed that enzymes are distributed in the cytoplasm regardless of the cell type or germ cell maturation stage. Although there was a steady distribution of enzymes across the seminiferous epithelium cycle, our experiments showed that spermatozoa from caput showed a higher intensity of H_2_S-releasing enzymes than more mature caudal spermatozoa (Fig. 4a). Spermatozoa obviously lose their H_2_S-releasing enzymes during passage through the epididymis. An interesting finding was made by a study describing the importance of H_2_S production for sperm quiescence in the rat epididymis^17^. In contrast to that in spermatozoa, the expression of CBS and CTH was increased towards the cauda epididymis^17^. Based on recent knowledge, the epididymal epithelium appears to compensate for sperm H_2_S-releasing enzyme loss during spermatozoa passage through the epididymis by increasing the self-production of enzymes. Nevertheless, the loss of H_2_S-releasing enzymes continues beyond the epididymis and is found in further steps of sperm maturation, capacitation and sperm-*zona pellucida* binding (Fig. 5d,e,f).

The highlights of our study were the detection of enzymatic production of H_2_S and its consequences in the form of –(S)_n_H in mammalian spermatozoa. We detected H_2_S in the sperm flagella of mice (Fig. 4e), humans (Fig. 5c), and boar (Fig. 5d). Moreover, we immunodetected all H_2_S-releasing enzymes in all models used in sperm flagellum; therefore, we indirectly related endogenous H_2_S production to its enzymes. Subsequently, we followed –(S)_n_H as a major result of H_2_S action. To the best of our knowledge, for the first time, we described and characterized proteins that underwent –(S)_n_H of cysteine in spermatozoa. Protein persulfidation (–(S)_n_H) was strictly located in the sperm midpiece (Fig. 4f), which highly corresponds to H_2_S occurrence and the location of its enzymes. These observations are consistent with H_2_S properties; although H_2_S diffuses well across membranes, its short half-life, which lasts a few seconds, a maximum of minutes^18^, does not allow it to sufficiently affect proteins over long distances. To further elucidate the role of –(S)_n_H in sperm physiology, we detected –(S)_n_H depending on the live/dead status of human spermatozoa (Fig. 6). Surprisingly, live and dead spermatozoa did not differ from each other in terms of –(S)_n_H (Fig. 6a,b). Because cell death is accompanied by a decrease in pH, H_2_S could be released from pH unstable iron–sulfur complexes located in mitochondria, thereby maintaining the –(S)_n_H of nearby proteins even after cell death. To label –SH and –(S)_n_H, we used the affinity of iodoacetamidofluorescein (IAF) to the –SH group. IAF was previously used in a study that addresses the quality of cryopreserved bull spermatozoa depending on –SH content^19^. Based on IAF staining, the researchers distinguished several patterns, whose distribution was dependent on sperm viability. Spermatozoa labelled strictly in the midpiece were associated with higher viability than spermatozoa labelled along its entire length, as was the case in our study. Interestingly, the pattern associated with viable spermatozoa is strikingly similar to the pattern of –(S)_n_H. It is possible that viable spermatozoa contain –(S)_n_H in their midpiece instead of free –SH, as was previously suggested^19^. If so, –(S)_n_H located specifically in the mitochondrial sheath may play an important role in sperm metabolism and redox defence. We supported this statement by identifying –(S)_n_H using mass spectrometry. In most cases, the identified proteins were associated with mitochondrial metabolism and flagellar movement (Fig. 6e). Some of these proteins have been reported to undergo –(S)_n_H, including glyceraldehyde-3-phosphate dehydrogenase, tubulin^16^ and l-lactate dehydrogenase^4^, but we were the first to observe that these proteins were persulfidated in human spermatozoa. Some persulfidation targets were previously discovered as S-nitrosylated, including A-kinase anchor protein, heat shock protein and semenogelin^10^, which supports the finding that S-nitrosylation serves as a –(S)_n_H precursor^4,5,20^. All these results prove that spermatozoa contain many proteins containing reactive cysteine through which proteins can be easily turned on and off by redox PTMs.

Spermatozoa are completely dependent on previously produced proteins once they leave the male reproductive tract; therefore, they are vulnerable to oxidative stress. Persulfidation could play a key role in this context because it prevents cysteine hyperoxidation and thus stops redox signalling pathway disruption and protein damage. The reducing abilities of H_2_S could be essential during sperm capacitation. As is known, capacitation is enhanced by reactive oxygen and nitrogen species, but overproduction of these reactive species leads to oxidative stress and cell death^1,21,22^. However, H_2_S, through persulfidation, could contribute to the maintenance of redox balance, and thus prevent premature capacitation, which is an often problem of sperm manipulation in vitro conditions (*e.g.* cell maintenance in vitro, cryopreservation). In this study, we provided evidence for the enzymatic production of H_2_S not only in the testis but also in spermatozoa. We detected CBS, CTH and MPST in mammalian spermatozoa and thus indirectly linked H_2_S with its enzymes. We visualized H_2_S and therefore were able to localize it to the sperm flagella, where it affects nearby proteins by persulfidation. We identified some persulfidated proteins seemingly crucial for sperm viability, and we outlined the impact of endogenous H_2_S production on male reproduction. We proved the existence of H_2_S-releasing enzymes, H_2_S, and persulfidation and considered the link between them in spermatozoa. Obviously, other sophisticated models of in vitro pharmacological treatment of sperm and/or targeted silencing of all H_2_S-releasing enzymes in somatic cells are needed for the achievement of experimental data leading to a comprehensive acknowledgement of H_2_S in the physiology of reproduction. Moreover, there is no doubt that H_2_S is an important signalling molecule that purposeful modulation deserves a knowledge transfer to different medical disciplines.

## Methods

All chemicals were purchased from Sigma Aldrich unless specified otherwise. Peanut agglutinin from *Arachis hypogaea* (PNA) conjugated with Alexa Fluor™ 488 was purchased from Thermo Fischer Scientific (MA, USA, #L21409). The primary polyclonal antibodies anti-cystathionine β-synthase (anti-CBS), anti-cystathionine γ-lyase (anti-CSE) and anti-3-mercaptopyruvate sulfurtransferase (anti-3MPST) as well as the secondary antibody goat anti-rabbit-Alexa Fluor^®^ 647 (# ab150079) were purchased from Abcam (Cambridge, UK).

### Animals, samples, and ethical statements

C57Bl/6 male mice aged 21 days and 12–14 weeks as well as all applied protocols as noted below were used in animal experiments, in accordance with the Protection of Animals against Cruelty (Act No. 246/1992 Coll.) of the Czech Republic and under the supervision of the Animal Welfare Advisory Committee at the Ministry of Education, Youth, and Sports of the Czech Republic (approval number: MSMT-249/2017-4). Alternatively, boar ejaculates were purchased from Chovservis Co. (Hradec Kralove, Czech Republic). Reports concerning experimental animals follow the recommendations in the ARRIVE guidelines^23^. Human semen samples were obtained after informed consent at the IVF Zentren Prof. Zech—Pilsen, Ltd. (Pilsen, Czech Republic); the study of human sperm was approved by the Ethics Committee of Charles University, Faculty of Medicine in Pilsen (238/2016). All methods were carried out in accordance with relevant guidelines and regulations (WHO manual 2010^24^).

### Preparation of sperm samples

Mouse spermatozoa isolated from the cauda epididymis were allowed to swim out to human tubal fluid medium with HEPES (HTF-HEPES, LifeGlobal™, LifeGlobal Group, USA). Ejaculated boar spermatozoa were diluted with Beltsville Thaw Solution (BTS) at a concentration of 10 mil/ml and stored at 18 °C for 2 days until utilization for the *zona pellucida*-binding assay. The rest of the boar spermatozoa were washed and resuspended in modified Tyrode's lactate-HEPES medium (TL-HEPES)^25^ at a concentration of 10 mil/ml. For capacitation, spermatozoa were resuspended in capacitated modified TL-HEPES medium^25^ at a concentration of 10 mil/ml and allowed to capacitate for 4 h at 37 °C. Capacitated spermatozoa were then washed from the capacitating medium and resuspended in noncapacitating modified TL-HEPES. Human ejaculates, obtained from three normozoospermic aged 30–35, were processed according to the WHO manual 2010^24^. Briefly, ejaculates were divided into the noncapacitated and capacitated groups. Spermatozoa were allowed to swim up from ejaculates into HTF-HEPES medium, which was placed over the ejaculate, for 2.5 h in a 37 °C water bath. In the case of the capacitated group, HTF-HEPES medium was enriched with 0.3% bovine serum albumin (BSA). Thereafter, all samples were processed according to the purpose stated below.

### Porcine *zona pellucida*-binding assay

Pig oocytes were obtained from ovaries of 6- to 8-month-old noncycling gilts (a crossbreed of Landrace × Large White), yielded at the slaughterhouse (Jatky Český Brod a.s., Český Brod, Czech Republic). First, cumulus-oocyte complexes were collected from ovarian follicles with a diameter of 2–5 mm by aspiration with a 20-gauge needle and handled in TL-HEPES-medium supplemented with 0.1 mg/ml polyvinyl alcohol (PVA). Immature oocytes were matured in vitro in modified tissue culture medium (mTCM; Gibco, Life Technologies, UK), as described earlier^26^. After 44 h of culture, cumulus cells were removed with 0.1% hyaluronidase, and matured oocytes with extruded polar bodies were selected for the binding assay. Spermatozoa stored in BTS medium were washed and resuspended in modified Tris-buffered medium (mTBM; Abeydeera et al., 1998) at a concentration of 1 mil/ml. Subsequently, 100,000 spermatozoa were added to oocyte-free zonas and coincubated in 0.5 ml of mTBM at 39 °C and CO_2_ for 30 min. Thereafter, *zona pellucida*-bound spermatozoa were washed in PBS supplemented with PVA, fixed in 4% paraformaldehyde (PFA) enriched with 0.1% Triton TX-100 and 1 mM DTT for 15 min at 37 °C, washed and stored in PBS with sodium azide at 4 °C for immunocytochemistry.

### Immunocytochemistry

Mouse, boar, and human spermatozoa were fixed and stored as described above. Then, spermatozoa were allowed to adhere to polylysine-coated coverslips, permeabilized with 0.1% Triton TX-100 for 40 min, and blocked with 0.1% Triton TX-100–10% normal goat serum (NGS)-1% BSA for 1 h at 37 °C. Subsequently, they were incubated with anti-CBS, anti-CSE and anti-3MPST antibodies diluted 1:100 at 4 °C overnight. Thereafter, coverslips were washed, followed by incubation with secondary antibody diluted 1:200 for 40 min at room temperature. PNA diluted 1:200 was added to the secondary antibody to follow the acrosome reaction. Coverslips were washed and mounted in Vectashield^®^ medium with 4′6′-diamino-2-phenylindole (DAPI; Vector Laboratories, Inc., CA, USA). Images were acquired using an Olympus IX83 fluorescence microscope (Olympus, Germany) and VisiView^®^ software (Visitron Systems GmbH, Germany).

### Immunofluorescence of mouse testes

Mouse testes were fixed in 4% PFA, embedded in paraffin wax with random orientation, and sectioned completely into 10-μm-thick slides. After deparaffinization, antigen retrieval was performed using preheated citrate buffer (pH 6.0). Thereafter, cross-sections were permeabilized with 0.1% Triton TX-100 for 40 min and blocked with 0.1% Triton TX-100–10% NGS-1% BSA for 1 h at 37 °C. Subsequently, they were incubated overnight at 4 °C with antibodies at the following dilutions: anti-CBS: 1:250, anti-CTH: 1:125, and anti-3-MPST: 1:150. In the case of CBS and CTH, slides were incubated with the preadsorbed secondary antibodies anti-rabbit-Alexa Fluor^®^ 647 (1:200, Abcam, Cambridge, UK, # ab150083) and PNA. For 3-MPST detection, slides were incubated with biotin-conjugated goat anti-rabbit antibody (1:200, # ab6720) for 40 min, washed and incubated with a cocktail of Alexa Fluor^®^ 647-conjugated streptavidin (1:500, Bioss, USA, # bs-0437R-A647) and PNA. Subsequently, the slides were washed, mounted and visualized as described above.

### Probe detection of H_2_S in spermatozoa

Epididymal mouse, ejaculated boar and human spermatozoa were resuspended in HTF-HEPES and TL-HEPES media, respectively, at a concentration of 2 mil/ml. Working solutions were prepared with adequate medium containing 500 µM acetyl trimethylammonium bromide (CTAB) and Sulfane Sulfur Probe 4 (SSP4) (SulfoBiotics, Dojindo EU GmbH, Munich, Germany) dissolved in DMSO at a concentration of 1:500. In the negative control, the SSP4 probe was omitted, and DMSO at the same concentration as SSP4 was used. For a positive control, spermatozoa were coincubated with pyridoxal-5′-phosphate (PxP) at a concentration of 50 mM for 30 min before incubation with the SSP4 probe. Subsequently, 200 µl of working solution and 5 µl of sperm suspension were added to polylysine-coated coverslips and incubated for 15 min at 37 °C, followed by slide mounting in PBS with Hoechst 33352 (1:1000`Sigma-Aldrich, MO, USA) and immediate evaluation.

### Colorimetric detection of H_2_S in testicular tissue

The enzymatic capacity to release H_2_S was assessed using a colorimetric approach as described earlier^27^, with slight modifications. Mouse testicular tissue was homogenized in extraction buffer (1% Zin(OAc)_2_, 20 mM EDTA, 50 mM Tris–HCl, pH 8), enriched with Complete Mini Protease Inhibitor Cocktail^®^ (Roche, Basel, Switzerland), and lysed for 20 min on ice. After centrifugation, the lysate was incubated with 2 mM PxP and 10 mM l-cysteine for 2 h at 37 °C in a N_2_ atmosphere. Lysates without PxP and/or l-cysteine, a cofactor of enzymes (CBS, CTH) and enzyme (CBS, CTH, MPST) substrate, respectively, were used as negative controls. Thereafter, proteins were precipitated with 12.5% trichloroacetic acid for 10 min, and the reaction was centrifuged. To 100 µl of the supernatant, 100 µl of 20 mM DMPE and 100 µl of FeCl_3_ were added and incubated for 10 min, and the absorbance was read at 670 nm. The absorbance was recalculated based on the standard curve of Na_2_S.9H_2_O, an exogenous H_2_S donor, and is expressed as nM H_2_S.mg of tissue^−1^ min^−1^.

### Western blot

Mouse testicular tissue, epididymal mouse spermatozoa and ejaculated boar and human spermatozoa were washed two times with TBS, and the pellets were dissolved in RIPA lysis buffer^28^ with 100 mM DTT, enriched with Complete Mini Protease Inhibitor Cocktail (Roche, Switzerland) and incubated for 30 min on ice. Thereafter, samples were subjected to sodium dodecyl sulfate polyacrylamide gel electrophoresis (SDS-PAGE) on a 4–15% separating Mini-PROTEAN^®^ precast gel and blotted using a Trans-Blot Turbo Transfer System onto PVDF membranes (Bio-Rad Laboratories, France). The membranes were blocked in 5% BSA in TBS with 0.05% Tween-20 (TBS-T) for 60 min at room temperature. The membrane was incubated with primary antibodies as mentioned above and diluted 1:1000 in 1% BSA in TBS-T overnight at 4 °C. A rabbit monoclonal anti-histone H3 antibody (1:1000, Abcam, Cambridge) was used as the internal control. Horseradish peroxidase-conjugated anti-rabbit IgG antibody (1:15,000; Invitrogen, Carlsbad, CA, USA) was applied for 60 min at room temperature. Target proteins were visualized using ECL Select Western Blotting Detection Reagent^®^ (GE Healthcare Life Sciences, UK) and a ChemiDoc^®^ MP System (Bio-Rad).

### 6-Iodoacetamidofluorescein (6-IAF) switch assay

Persulfidated proteins were visualized in spermatozoa using a modified switch assay. First, spermatozoa were subjected to a LIVE/DEAD Fixable Dead Cell Stain Kit (Invitrogen Life Technologies, Carlsbad, CA, USA, #L23105) as previously described^29^. Persulfidation (–(S)_n_H) and free thiols (–SH) were distinguished in accordance with^16^. Briefly, free thiols of spermatozoa were blocked by 20 mM methyl methanethiosulfonate (MMTS, Sigma-Aldrich, MO, USA, #64306) dissolved in HEN buffer (250 mM HEPES–NaOH (pH 8), 1 mM EDTA, and 0.1 mM neocuproine) for 60 min at 38 °C on a shaker. After blocking, the sperm suspension was washed three times with PBS for 10 min on a shaker and centrifuged (300 g). Presumed –(S)_n_H in spermatozoa was stained with 0.04 μM 6-iodoacetamidofluorescein (6-IAF, Thermo Fisher, USA, #I30452) for 15 min at room temperature and fixed in 3.2% PFA for 10 min. The prepared samples were analysed using a BD FACS Aria fusion cell analyser (Becton Dickinson, Prague, Czech Republic) for flow cytometry. Data were collected from 5000 events. LIVE/DEAD Fixable Dead Cell Stain and 6-IAF were excited by 405 and 488 nm lasers and detected with 450/50 and 530/30 bandpass filters. Acquired data were analysed using FlowJo software (Becton Dickinson, Prague, Czech Republic). Alternatively, spermatozoa were settled down to coverslips, and –(S)_n_H was visualized in situ via an Olympus IX83 fluorescence microscope (Olympus, Germany).

### Biotin switch method and pulldown assay of human sperm and mouse testis, liver and brain

Detection of –(S)_n_H in lysate was processed as previously described^16^ with slight modifications. Briefly, tissues were lysed in 100 μL of HENS buffer (250 mM HEPES–NaOH (pH 8), 1 mM EDTA, and 0.1 mM neocuproine, 1% SDS) and incubated on a shaker for 30 min. Then, lysates were centrifuged (10,000 g), 50 μL of supernatant was mixed with 150 μL of HEN buffer, and 0.38 μL of MMTS was added (reaching a final concentration of 20 mM). Free thiols in protein lysate were blocked for 20 min at 50 °C on a shaker. The residue of MMTS was then removed by ethyl acetate extraction, vortexed three times followed by brief centrifugation and ethyl acetate removal by pipette, followed by vacuum evaporation. The samples were labelled with the final concentration 3.3 mM EZ-linked iodoacetyl-PEG_2_-biotin (Thermo Fisher, USA; #21334) overnight at 4 °C on a shaker. An aliquot of treated proteins was diluted in Laemmli loading buffer under reducing agent-free conditions; samples were separated by SDS-ELFO and visualized by Western blotting as described above using HRP-conjugated streptavidin (1:1000; Sigma-Aldrich, MO, USA; #18-152) and chemiluminescence detection as described previously. Alternatively, lysates were loaded onto streptavidin-coated agarose beads (Millipore, MA, USA; #16-126) and incubated overnight at 4 °C on a shaker. Beads were treated with 100 mM β-mercaptoethanol in 4% SDS, and primary persulfidated proteins were eluted. The purified samples were processed for nano-LC–MS as described below.

### Nano-LC–MS

Tissue lysates from animals and human spermatozoa were used for complete proteomic analysis. Nanoliquid chromatography-MS (nano-LC–MS) was used for protein identification and quantification, as described previously^30^.

### Statistics

Data were analysed using GraphPad Prism 8 (GraphPad Software, Inc., San Diego, CA, USA). Based on Shapiro–Wilks normality distribution tests, differences were tested as noted below. *P* values ≤ 0.05, 0.01, 0.001, and 0.0001 were considered statistically significant and are indicated with asterisks (*), (**), (***), and (****), respectively.

## Supplementary Information

Supplementary Information.

## References

[1] Aitken RJ (2017). Reactive oxygen species as mediators of sperm capacitation and pathological damage. Mol. Reprod. Dev..

[2] Machado-Oliveira G (2008). Mobilisation of Ca^2+^ stores and flagellar regulation in human sperm by S-nitrosylation: A role for NO synthesised in the female reproductive tract. Development.

[3] O’Flaherty C (2015). Redox regulation of mammalian sperm capacitation. Asian J. Androl..

[4] Fu L (2020). Direct proteomic mapping of cysteine persulfidation. Antioxid. Redox Signal..

[5] Longen S (2016). Quantitative Persulfide Site Identification (qPerS-SID) reveals protein targets of H2S releasing donors in mammalian cells. Sci. Rep..

[6] Wood ZA, Schröder E, Harris JR, Poole LB (2003). Structure, mechanism and regulation of peroxiredoxins. Trends Biochem. Sci..

[7] Sun J, Steenbergen C, Murrhy E (2006). S-Nitrosylation: NO-related redox signaling to protect against oxidative stress. Antioxid. Redox Signal..

[8] Dóka (2020). Control of protein function through oxidation and reduction of persulfidated states. Sci. Adv..

[9] Petrovic D, Kouroussis E, Vignane T, Filipovic MR (2021). The role of protein persulfidation in brain aging and neurodegeneration. Front. Aging Neurosci..

[10] Lefièvre L (2007). Human spermatozoa contain multiple targets for protein S-nitrosylation: An alternative mechanism of the modulation of sperm function by nitric oxide?. Proteomics.

[11] Li G, Xie ZZ, Chua JMW, Wong PC, Bian J (2015). Hydrogen sulfide protects testicular germ cells against heat-induced injury. Nitric Oxide Biol. Chem..

[12] Wang J (2017). Hydrogen sulfide as a potential target in preventing spermatogenic failure and testicular dysfunction. Antioxid. Redox Signal..

[13] Zhang Y (2013). Hydrogen sulfide, the next potent preventive and therapeutic agent in aging and age-associated diseases. Mol. Cell. Biol..

[14] Pintus E, Jovičić M, Kadlec M, Ros-Santaella JL (2020). Divergent effect of fast- and slow-releasing H2S donors on boar spermatozoa under oxidative stress. Sci. Rep..

[15] Zhang W (2018). Decrease in male mouse fertility by hydrogen sulfide and/or ammonia can Be inheritable. Chemosphere.

[16] Mustafa AK (2009). HS signals through protein S-Sulfhydration. Sci. Signal..

[17] Gao DD (2019). Cellular mechanism underlying hydrogen sulfide mediated epithelial K^+^ secretion in rat epididymis. Front. Physiol..

[18] Polhemus DJ, Lefer DJ (2014). Emergence of hydrogen sulfide as an endogenous gaseous signaling molecule in cardiovascular disease. Circ. Res..

[19] Chatterjee S, de Lamirande E, Gagnon C (2001). Cryopreservation alters membrane sulfhydryl status of bull spermatozoa: Protection by oxidized glutathione. Mol. Reprod. Dev..

[20] Finelli MJ (2020). Redox post-translational modifications of protein thiols in brain aging and neurodegenerative conditions—Focus on S-Nitrosation. Front. Aging Neurosci..

[21] Aitken RJ (2011). The capacitation-apoptosis highway: Oxysterols and mammalian sperm function. Biol. Reprod..

[22] O’Flaherty C, Matsushita-Fournier D (2017). Reactive oxygen species and protein modifications in spermatozoa†. Biol. Reprod..

[23] Kilkenny C, Browne WJ, Cuthill IC, Emerson M, Altman DG (2010). Improving bioscience research reporting: The arrive guidelines for reporting animal research. PLoS Biol..

[24] World Health Organization (2010). WHO Laboratory Manual for the Examination and Processing of Human Semen.

[25] Kerns K, Zigo M, Sutovsky P (2018). Zinc: A necessary ion for mammalian sperm fertilization competency. Int. J. Mol. Sci..

[26] Abeydeera LR, Wang WH, Cantley TC, Prather RS, Day BN (1998). Presence of β-mercaptoethanol can increased the glutathione content of pig oocytes matured in vitro and the rate of blastocyst development after in vitro fertilization. Theriogenology.

[27] Xu Z (2009). Ischemia-reperfusion reduces cystathionine-β-synthase-mediated hydrogen sulfide generation in the kidney. Am. J. Physiol. Renal Physiol..

[28] Zigo M, Manaskova-Postlerova P, Jonakova V, Kerns K, Sutovsky P (2019). Compartmentalization of the proteasome-interacting proteins during sperm capacitation. Sci. Rep..

[29] Ruiz-Díaz S (2020). Changes in the cellular distribution of tyrosine phosphorylation and its relationship with the acrosomal exocytosis and plasma membrane integrity during in vitro capacitation of frozen/thawed bull spermatozoa. Int. J. Mol. Sci..

[30] Nevoral J, Kolinko Y, Moravec J, Žalmanová T, Hošková K, Prokešová S (2018). Long-term exposure to very low doses of bisphenol S affects female reproduction. Reproduction.

