# Supplementary material for: Evidence of endogenously produced hydrogen sulfide (H2S) and persulfidation in male reproduction
Source: Sci Rep. 2022 Jul 6;12:11426. doi: 10.1038/s41598-022-15360-x (PMC9259693; doi:10.1038/s41598-022-15360-x)
Supplement: Supplementary file 1 — Supplementary Information. [file 41598_2022_15360_MOESM1_ESM.docx]

**Supplementary Informations**

Evidence of endogenously produced hydrogen sulfide (H_2_S) and persulfidation in male reproduction

Hedvika Řimnáčová, Jiří Moravec, Miriama Štiavnická, Jiřina Havránková, Ladan Monsef, Petr Hošek, Šárka Prokešová, Tereza Žalmanová, Jaroslav Petr, Milena Králíčková, Jan Nevoral

**Content**

Supplementary figure S1: The phylogenetic relation of H_2_S-releasing enzymes

Supplementary figure S2: Whole PVDF membrane with detected H_2_S-releasing enzymes in mammalian sperm lysates.

Supplementary figure S3: The PVDF membrane with persulfidated proteins detected in sperm lysates.


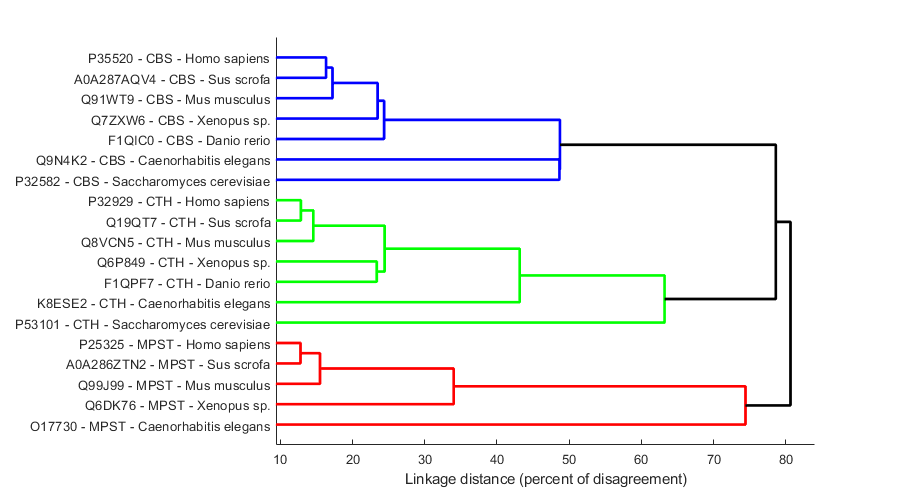


Supplemental figure S1: Phylogenetic tree based on amino acid sequences of H_2_S-releasing enzymes, cystathionine γ-lyase (CTH), cystathionine β-synthase (CBS), and 3-mercaptopyruvate sulfurtransferase (MPST), across the most frequently used model organisms, has underlined mammals' closest relation, especially pig and human (CBS: blue, CTH: green, MPST: red). Phylogenetic tree was created by hierarchical clustering based on the single linkage rule from a similarity matrix imported in MATLAB (version 2019b, The MathWorks, Inc., Natick, MA, USA).


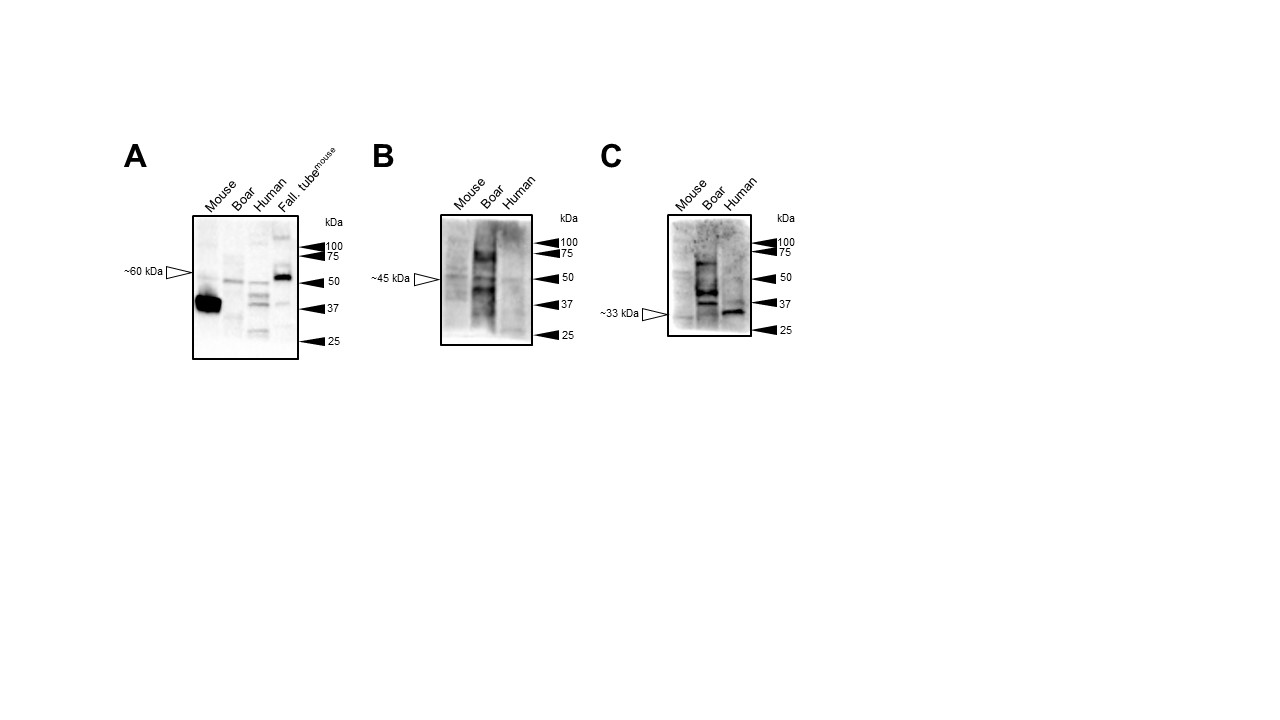


Supplemental figure S2: Whole PVDF membrane with detected H_2_S-releasing enzymes in mammalian sperm lysates. (A) CBS, (B) CTH, and (C) MPST immunoreactive bands in mouse, boar, and human sperm lysates. Empty arrowheads indicate presumed band belonging to CBS, CTH, and MPST, respectively. Due to concerns about CBS antibody specify, fallopian tube (mouse) was loaded as a positive control, whilst ~60kDa band was proved due to MALDI-TOF.


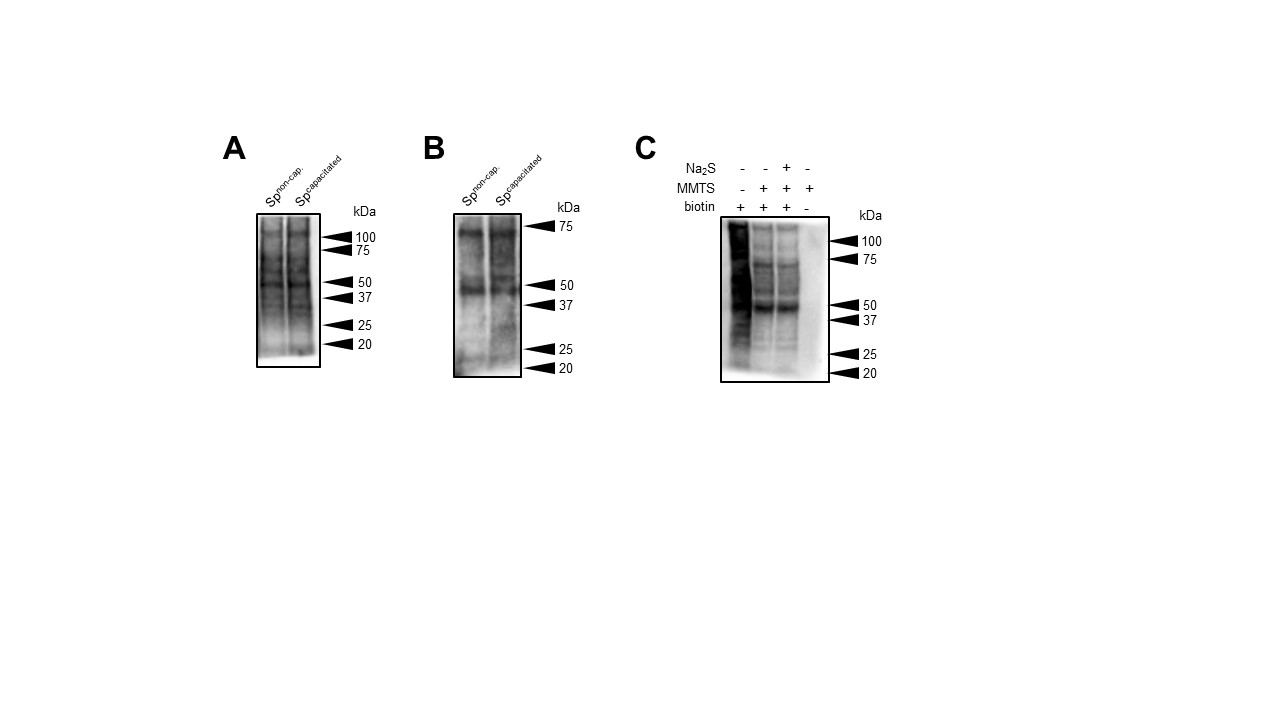


Supplemental figure S3: The PVDF membrane with persulfidated proteins detected in sperm lysates, using the biotin-labelling assay and western blot. (A) Persulfidation in non-capacitated and capacitated mouse spermatozoa. (B) Persulfidation in non-capacitated and capacitated boar spermatozoa. (C) Validation of biotin-labelling assay using boar sperm lysate. Positive control was prepared: free thiols blocking agents (MMTS) was omitted, whilst all thiols in lysate reacted with iodoacetyl-PEG_2_-biotin. The negative control, omitting biotin-labelled iodoacetyl-PEG_2_ and using iodoacetamide only. The treatment with 100μM Na_2_S .9H_2_O, an exogenous H_2_S donor, was tested for the induction of persulfidation in sperm lysates.
